# A randomized clinical trial of bermekimab treatment for clinical improvement of systemic sclerosis

**DOI:** 10.1016/j.isci.2023.107670

**Published:** 2023-08-19

**Authors:** Nicky Solomonidi, Panayiotis G. Vlachoyiannopoulos, Maria Pappa, Georgia Liantinioti, Sofia Ktena, Evangelos Theotikos, Antonia Elezoglou, Mihai G. Netea, Evangelos J. Giamarellos-Bourboulis

**Affiliations:** 1 4th Department of Internal Medicine, National and Kapodistrian University of Athens, Medical School, Athens, Greece; 2 Department of Pathophysiology, National and Kapodistrian University of Athens, Medical School, Athens, Greece; 3 Department of Rheumatology, Asklepieion General Hospital of Voula, Athens, Greece; 4 Department of Internal Medicine and Center for Infectious Diseases, Radboud University, Nijmegen 6500, the Netherlands; 5 Department of Immunology and Metabolism, Life and Medical Sciences Institute, University of Bonn, Bonn, Germany

**Keywords:** Health sciences, Medicine, Neurology, Pharmacology

## Abstract

Increased concentrations of interleukin (IL)-1α have been recently described in tissues of patients with systemic sclerosis (SSc) suggesting that IL-1α inhibition may be a target for treatment. We conducted a double-blind, placebo-controlled study to assess the safety and efficacy of the fully humanized IL-1α blocking monoclonal antibody bermekimab in SSc. To evaluate response to treatment, we developed the score of inhibition of progression of SSc which was validated using the CRISS index and the modified CRISS index. The primary endpoint was met in 80% of bermekimab-treated patients vs. 20% of placebo-treated patients (p: 0.023). Most of efficacy was found for increase of carbon monoxide lung diffusion capacity. Production of IL-1α and TNF by circulating mononuclear cells was decreased and the absolute count of CD42/Cd62-platelets was decreased. Results suggest that bermekimab is a promising treatment for SSc.

## Highlights

• Bermekimab is a monoclonal antibody blocking the activity of IL-1α
• 12-week treatment provides global improvement in systemic sclerosis
• Decrease of inflamed joints and improvement of lung function prevail
• Production of IL-1α and TNF by circulating monocytes is decreased

## Graphical abstract

**Figure:**
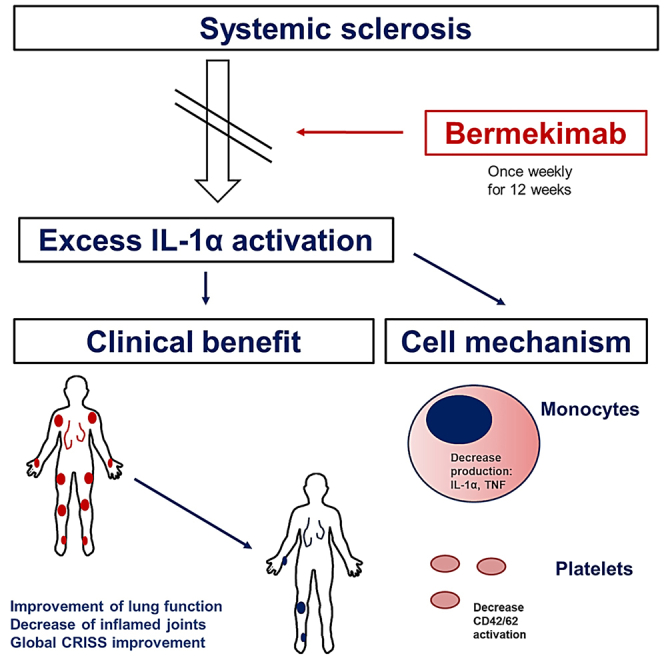

## Introduction

Effective treatment of systemic sclerosis (SSc) remains an unmet medical need. Fibrosis and vasculopathy affecting the skin, lungs, gastrointestinal tract, kidneys, and heart is the hallmark of SSc and causes functional difficulties that handicap quality of life and elicit high morbidity and mortality.^1^^,^^2^^,^^3^^,^^4^^,^^5^

Recent evidence suggests that the pro-fibrotic action in SSc is mediated at least in part through over-production of interleukin (IL)-1α. Circulating IL-1α is higher among patients than healthy volunteers,^6^^,^^7^ and IL-1α is over-produced from the fibroblasts of the patients. IL-1α bounds with higher affinity than IL-1β to the active IL-1R1 receptor on fibroblasts and elicits a vicious cycle of fibroblast activation and production of pre-collagen.^8^ IL-1α is also bridging platelets to vascular endothelium and mediates vasculopathy of SSc.^9^

Bermekimab is a human antibody targeting and blocking the bioactivity of IL-1α, previously studied in colorectal and metastatic cancer as well as in hidradenitis suppurativa (HS).^10^^,^^11^^,^^12^ Treatment with bermekimab decreased tumor outgrowth probably through inhibition of vascular microthrombi,^10^^,^^11^ and attenuated inflammation in HS patients. Its efficacy was associated with modulation of the production of human β-defensin-2 (hBD-2) by circulating neutrophils.^12^ Fibrosis evidenced by scars of the affected areas and by skin ultrasound is one main complications of HS^13^^,^^14^: therefore, favorable outcomes of bermekimab in HS generate hopes for similar efficacy in SSc.

The LIGHT trial (ClinicaL efficacy of Inhibition of orGan dysfunction througH bermekimab in systemic sclerosis: a proof-of-concept double-blind randomized clinical Trial) investigated the clinical efficacy of IL-1α inhibition in advanced SSc by bermekimab. Clinical efficacy was evaluated by the introduction of a novel composite primary endpoint merging endpoints of past clinical trials.

## Results

### Study population

**Figure 1:**
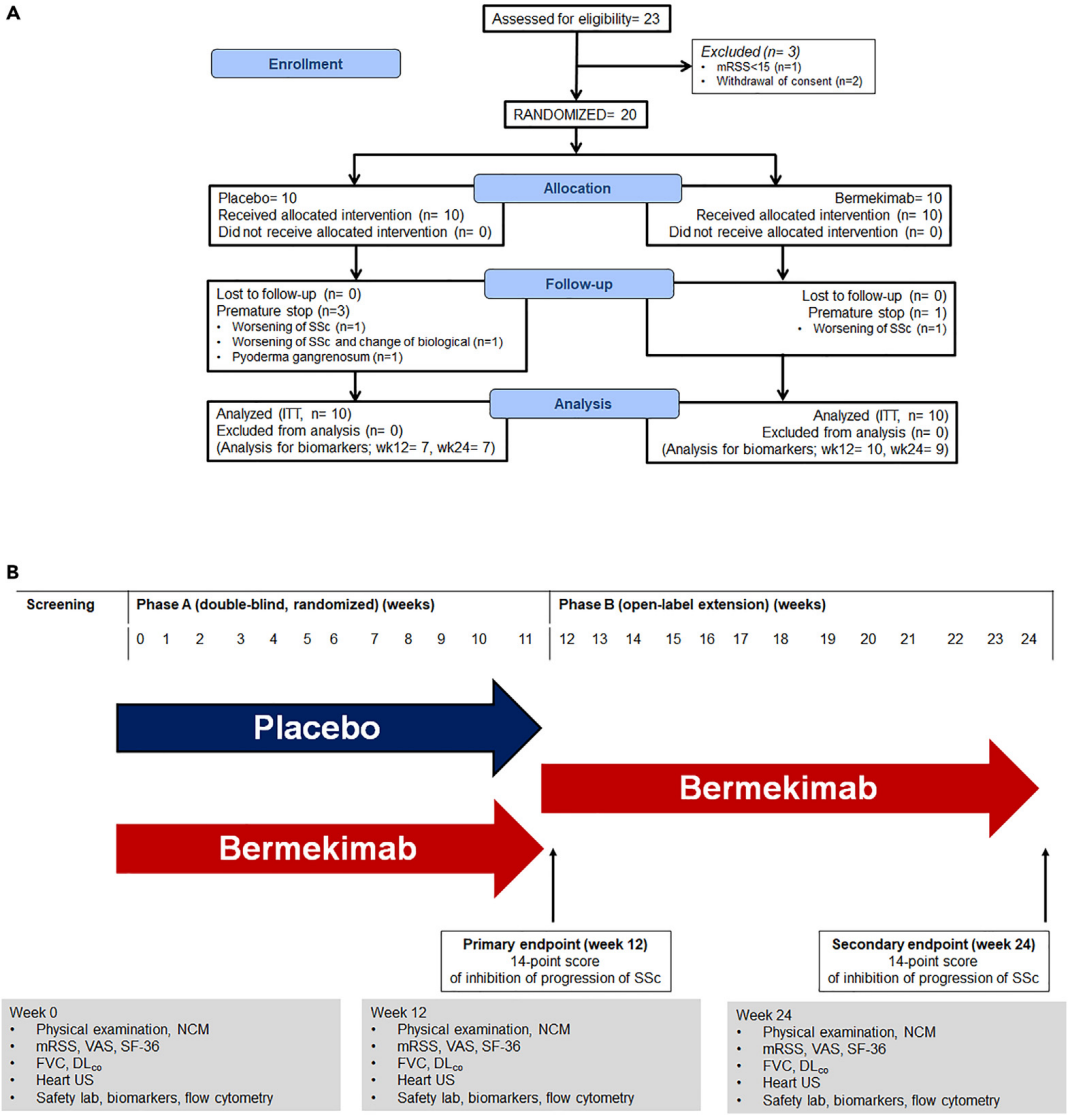
The LIGHT trial (see also Table S2) (A) Study flow chart. (B) Study design. Abbreviations DL_CO_: carbon monoxide diffusion capacity; FVC: forced vital capacity; ITT: intent-to-treat; mRSS, modified Rodnan skin score; NCM: nailfold capillaromiscroscopy; SSc: systemic sclerosis; SF-36: Short Form 36; VAS: visual analogue scale; wk: week.

The first patient was enrolled on September 10, 2019 and the last study visit of the last patient was on January 7, 2021. Patient demographics and characteristics are shown on Table 1 and on Table S1; the study flow chart is provided in Figure 1. From 23 screened patients three patients failed to be enrolled in the trial; two patients withdrew consent; and one patient did not meet inclusion criteria. As a consequence, 20 patients were randomized; no patient was lost to follow-up. The study drug was discontinued prematurely in four patients; in three patients due to progression of SSc; and in one patient due to the presentation of pyoderma gangrenosum.

**Table 1:** Baseline demographics and characteristics of enrolled patients

	Placebo (n = 10)	Bermekimab (n = 10)	p-value
Age, mean ± SD, years	56.5 ± 13.3	45.2 ± 13.2	0.072
Female gender, n (%)	10 (100)	8 (80)	0.474

**Comorbidities, n (%)**			

Type 2 diabetes mellitus	1 (10)	1 (10)	1.00
Chronic heart failure	2 (20)	0 (0)	0.474
Chronic obstructive pulmonary disease	0 (0)	3 (30)	0.211
Chronic intake of corticosteroids	5 (50)	5 (50)	1.00
Parkinson’s disease	1 (10)	0 (0)	1.00
Nephrolithiasis	1 (10)	0 (0)	1.00
Gallstones	1 (10)	0 (0)	1.00
Coronary heart disease	1 (10)	2 (20)	1.00
Atrial fibrillation	1 (10)	0 (0)	1.00
Depression	5 (50)	2 (20)	0.350

**Clinical findings, mean ± SD**			

Number of inflamed joints∗	8.10 ± 6.99	5.70 ± 3.86	0.355
Modified Rodnan Skin Score	20.20 ± 3.36	27.10 ± 8.93	0.035
Number of digital ulcers	0.90 ± 1.01	3.30 ± 2.94	0.027
Body mass index, kg/m^2^	27.98 ± 6.08	22.35 ± 5.43	0.043
Capillary density at NCM	2.40 ± 0.52	2.60 ± 0.69	0.476
Systolic blood pressure, mmHg	104.2 ± 33.6	120.2 ± 17.9	0.201
Diastolic blood pressure, mmHg	73.3 ± 12.2	80.6 ± 12.7	0.207
Heart rate,/min	74.8 ± 11.1	78.7 ± 11.1	0.453

**Skin findings, n (%)**			

Skin thickness of both hands proximal to MCPs	10 (100)	10 (100)	1.00
Puffy fingers	10 (100)	8 (80)	0.474
Sclerodactyly of the fingers	10 (100)	10(100)	1.00
Digital tip ulcers	8 (80)	8 (80)	1.00
Fingertip pitting scars	4 (40)	6 (60)	0.656
Telangiectasia	9 (90)	10 (100)	1.00
Abnormal nailfold capillaries	9 (90)	10 (100)	1.00
Pulmonary arterial hypertension	1 (10)	4 (40)	0.303
Interstitial lung disease	8 (80)	6 (60)	0.628
SSc related antibodies	9 (90)	10 (100)	1.00
Duration of SSc, median (range)	6.5 (0–20)	9.0 (2–25)	0.190

**Affected join areas, n (%)**			

Left shoulder	4 (40)	4 (40)	1.00
Left elbow	5 (50)	5 (50)	1.00
Left carpal	8 (80)	8 (80)	1.00
Left MCP	5 (50)	4 (40)	1.00
Left hip	1 (10)	1 (10)	1.00
Left knee	3 (30)	2 (20)	1.00
Left ankle	2 (20)	1 (10)	1.00
Right shoulder	4 (40)	4 (40)	1.00
Right elbow	5 (50)	6 (60)	1.00
Right carpal	8 (80)	8 (80)	1.00
Right MCP	4 (40)	4 (40)	1.00
Right hip	1 (10)	1 (10)	1.00
Right knee	3 (30)	2 (20)	1.00
Right ankle	2 (20)	2 (20)	1.00

**Musculoskeletal manifestations, n (%)**			

Arthalgia	10 (100)	8 (80)	0.474
Synovitis	3 (30)	3 (30)	1.00
Contractures	7 (70)	8 (80)	1.00
Tendon friction rubs	3 (30)	2 (20)	1.00
Tenosynovitis	0 (0)	1 (10)	1.00
Myalgia	7 (70)	4 (40)	0.370
Muscle weakness	8 (80)	7 (70)	1.00
**Skin manifestations, n (%)**			
Digital ulcers and calcinosis	7 (70)	8 (80)	1.00

**Gastrointestinal manifestations n (%)**			

Dysphagia	5 (50)	7 (70)	0.650
Heartburn	6 (60)	9 (90)	0.303
Bloating	5 (50)	3 (30)	0.650
Abdominal pain	4 (40)	5 (50)	1.00
Nausea	3 (30)	1 (10)	0.582
Vomiting	3 (30)	0 (0)	0.211
Diarrhea	4 (40)	2 (20)	0.628
Constipation	2 (20)	1 (10)	1.00

**Lung manifestations, n (%)**			

Cough	3 (30)	3 (30)	1.00
Limited exercise tolerance	5 (50)	8 (80)	0.350
Dyspnea	6 (60)	5 (50)	1.00

**Quality of life scores, mean ± SD**			

UCLA GIT score, points	7.83 ± 5.94	5.22 ± 4.60	0.355
Global VAS for SSc, mm	59.0 ± 29.2	52.0 ± 19.9	0.539
VAS for fatigue, mm	64.0 ± 27.9	53.0 ± 33.4	0.435
VAS for dyspnea, mm	49.0 ± 28.1	44.0 ± 22.2	0.664
SF-36 questionnaire, points	93.1 ± 4.5	95.4 ± 7.9	0.437

**Laboratory baseline findings**			

White blood cells,/mm^3^, mean ± SD	6522.5 ± 1008.7	6333.3 ± 2321.6	0.839
% neutrophils, mean ± SD	65.3 ± 10.6	59.4 ± 9.4	0.297
% lymphocytes, mean ± SD	24.4 ± 11.5	28.6 ± 6.9	0.440
% monocytes, mean ± SD	6.96 ± 2.49	9.13 ± 3.71	0.214
Creatinine, mean ± SD	0.66 ± 0.11	0.60 ± 0.13	0.260
Alanine aminotransferase, U/l, mean ± SD	17.5 ± 8.9	18.5 ± 12.4	0.863
Total bilirubin, mg/dl, mean ± SD	0.46 ± 0.18	0.41 ± 0.10	0.448
International normalized ratio, mean ± SD	1.00 ± 0.10	0.98 ± 0.07	0.455
DL_CO_, mean ± SD	76.6 ± 18.1	73.2 ± 17.5	0.699
Forced vital capacity, mean ± SD	77.0 ± 21.2	75.4 ± 17.2	0.870
Left ventricular ejection fraction, %, mean ± SD	58.6 ± 11.1	60.5 ± 3.7	0.629
Pulmonary arterial pressure, mmHg, mean ± SD	31.0 ± 10.1	28.7 ± 7.7	0.594

**Prior medications, n (%)**			

Prednisolone	5 (50)	5 (50)	1.00
Methotrexate	2 (20)	3 (30)	1.00
Cyclophosphamide	3 (30)	4 (40)	1.00
Azathioprine	4 (40)	0 (0)	0.087
Denosumab	1 (10)	1 (10)	1.00

**Concomitant medications, n (%)**			

Prednisolone	6 (60)	4 (40)	0.656
Endothelin antagonists	3 (30)	5 (50)	0.650
Mycophenolate	3 (30)	3 (30)	1.00
Methotrexate	4 (40)	2 (20)	0.628
Sildenafil	4 (40)	3 (30)	1.00
Proton pump inhibitors	4 (40)	6 (60)	0.656
Furosemide	1 (10)	1 (10)	1.00
ACE inhibitors	1 (10)	1 (10)	1.00
Vasodilators	2 (20)	1 (10)	0.582

ACE, angiotensin-converting enzyme; DL_CO_, carbon monoxide diffusing capacity; MCP, metacarpophalangeal; NCM, nailfold capillaromicroscopy; SD, standard deviation; SF-36, short form 36 health survey; SSc, systemic sclerosis; VAS, visual analogue scale.

### Primary study endpoint

The primary endpoint of the LIGHT trial was the comparison of the rate of patients scoring positive for the score of inhibition of SSc between the placebo arm and the treatment arm. When the LIGHT trial was designed, the CRISS (clinical response index of diffuse SSc) index^15^ had not been introduced. We then considered that the primary endpoint to investigate the global efficacy of a new drug on SSc should contain as many information as possible for the function of all systems affected by SSc. To this end, we integrated in the score of inhibition of SSc all elements coming from the previous trials on what the elements of improvement of SSc are (Table S2). We then validated the performance of our score using the CRISS index and the revised CRISS index, which were introduced later.^15^^,^^16^

A score of inhibition of SSc progression 4 or more was achieved in 80% of bermekimab-treated patients (8 out of 10 patients) and in 20% of the placebo-treated patients (2 out of 10 patients) at week 12 (p = 0.023) (Table 2; Table S3). Forward stepwise logistic regression analysis including all variables which were different at baseline between the two groups of treatment showed bermekimab treatment to be the only variable favoring the achievement of the primary endpoint (Table S4).

**Table 2:** Primary and secondary endpoints at the end of phase A of the study

	Bermekimab (n = 10)	Placebo (n = 10)	OR (95% CIs)	p value
**Primary endpoint**				

Score of inhibition of SSc progression 4 or more, n (%)	8 (80)	2 (20)	16.00 (1.78–143.15)	**0.023**
CRISS index, median (Q1-Q3)^b^	0.99 (0.98–1.00)	0.01 (0.00–0.99)	N/A	**0.011**

**Secondary endpoints**				

Score of inhibition of SSc progression, median (Q1-Q3)	5 (3.5–6.2)	2.0 (0–3.3.5)	N/A	0.005
Positive elements of the score of inhibition of SSc progression, n (%)				
At least 30% decrease of the number of inflamed joints	8 (80)	3 (30)	9.33 (1.19–72.99)	0.070
At least 30% decrease of modified Rodnan Skin Score	2 (20)	1 (10)	2.25 (0.17–29.76)	1.00
At least 30% decrease of digital ulcers	5 (50)	3 (30)	2.33 (0.37–14.61)	0.650
At least 50% decrease of UCLA-GIT	3 (30)	1 (10)	3.85 (0.32–45.57)	0.582
At least 50% increase of the short form 36 health survey	3 (30)	4 (40)	1.56 (0.24–9.91)	1.00
At least 50% decrease of global VAS	2 (20)	0 (0)	a	
At least 50% decrease of VAS for fatigue	2 (20)	1 (10)	2.25 (0.17–29.76)	1.00
At least 50% decrease of VAS for dyspnea	2 (20)	2 (20)	1.00 (0.11–8.94)	1.00
Any increase of body mass index	4 (40)	1 (10)	6.00 (0.53–67.65)	0.303
At least 10% decrease of nailfold capillaromicroscopy	2 (20)	1 (10)	2.25 (0.17–29.76)	1.00
Any increase of carbon monoxide diffusing capacity	6 (60)	0 (0)	a	**0.011**
Any increase of forced vital capacity	5 (50)	2 (20)	4.00 (0.54–29.09)	0.350
At least 10% increase of left ventricular ejection fraction	1 (10)	1 (10)	1.00 (0.05–18.57)	1.00
At least 10% decrease of pulmonary arterial pressure	4 (40)	1 (10)	6.00 (0.53–67.65)	0.303

CRISS, clinical response index of diffuse SSc; SD, standard deviation; NA, not applicable; Q, quartile; SSc, systemic sclerosis; UCLA-GIT, University of California Los Angeles Gastrointestinal Tract scoring system; VAS, visual analogue scale.

a cannot be calculated because one value is zero.

b although not part of the study protocol, the CRISS index is provided for validation purposes of the score of inhibition of SSc progression.

We validated the performance of our score in two approaches. The first approach was to investigate if score achievers have CRISS index greater than non-achievers. The second approach was to compare CRISS index between patients treated with bermekimab and patients treated with placebo. In the overall LIGHT cohort, 10 patients achieved score of inhibition of SSc progression 4 or more at week 12; another 10 patients did not achieve the score. Their mean ± SD CRISS index was 0.88 ± 0.29 and 0.40 ± 0.59 (p: 0.019) respectively indicating that the CRISS index is greater among score achievers. Bermekimab treatment was accompanied by significantly higher CRISS index than placebo treatment at week 12 (Table 2). We also analyzed retrospectively the performance of the revised CRISS index to validate the improvement of patients under bermekimab. Results showed that the odds ratio for better outcome under bermekimab treatment was greater reaching 7.36 when the percentage of improvement was reaching 30% (Table S5).

### Secondary study endpoints

The main secondary study endpoints were the score of inhibition of the progression of SSc at weeks 12 and 24 and the impact of bermekimab treatment on each of the elements of the score at week 12. The median score of inhibition at week 12 was 5 among bermekimab-treated patients compared to 1 among placebo-treated patients, a statistically significant difference (p: 0.005). However, this score was not different between patients treated for 12-week and patients treated for 24-week with bermekimab (Table S6). This analysis shows that clinical benefit is achieved after the first 12 weeks of treatment and that this is maintained with prolonged 24-week treatment. More precisely, all eight patients who had positive score of inhibition of SSc progression at week 12 maintained this response at week 24. Median score of inhibition of SSc progression increased from 1 at week 12 to 4 at week 24 when patients originally allocated to placebo switched to bermekimab during the open-label extension (OLE) period (p: 0.048) (Table S5).

Further analysis showed that most of bermekimab benefit was found for the number of inflamed joints and for the lung function expressed by the carbon monoxide diffusion capacity (Table 2). A statistical trend was found for the forced vital capacity (FVC) to increase among patients treated with bermekimab (5 out of 10 patients compared to 2 out of 10 treated with placebo; p: 0.054 by the binomial test).

Not all 14 elements of the score of inhibition of SSc progression were captured after the end of the follow-up 24-week period of the trial. In order to investigate if treatment efficacy was maintained after 24 weeks, the following information was collected for each patient at weeks 36 and 48: (1) if the patient was alive without need for start of any new drug; and (2) if the patient was experiencing either subjective improvement or the same degree of joint involvement, fatigue and change of modified Rodnan skin score (mRSS) as in week 24. Patients meeting both above conditions were considered maintaining treatment efficacy. Results showed that the benefit of bermekimab was maintained until week 48 (Table S7; Figure S1).

### Exploratory endpoints

**Figure 2:**
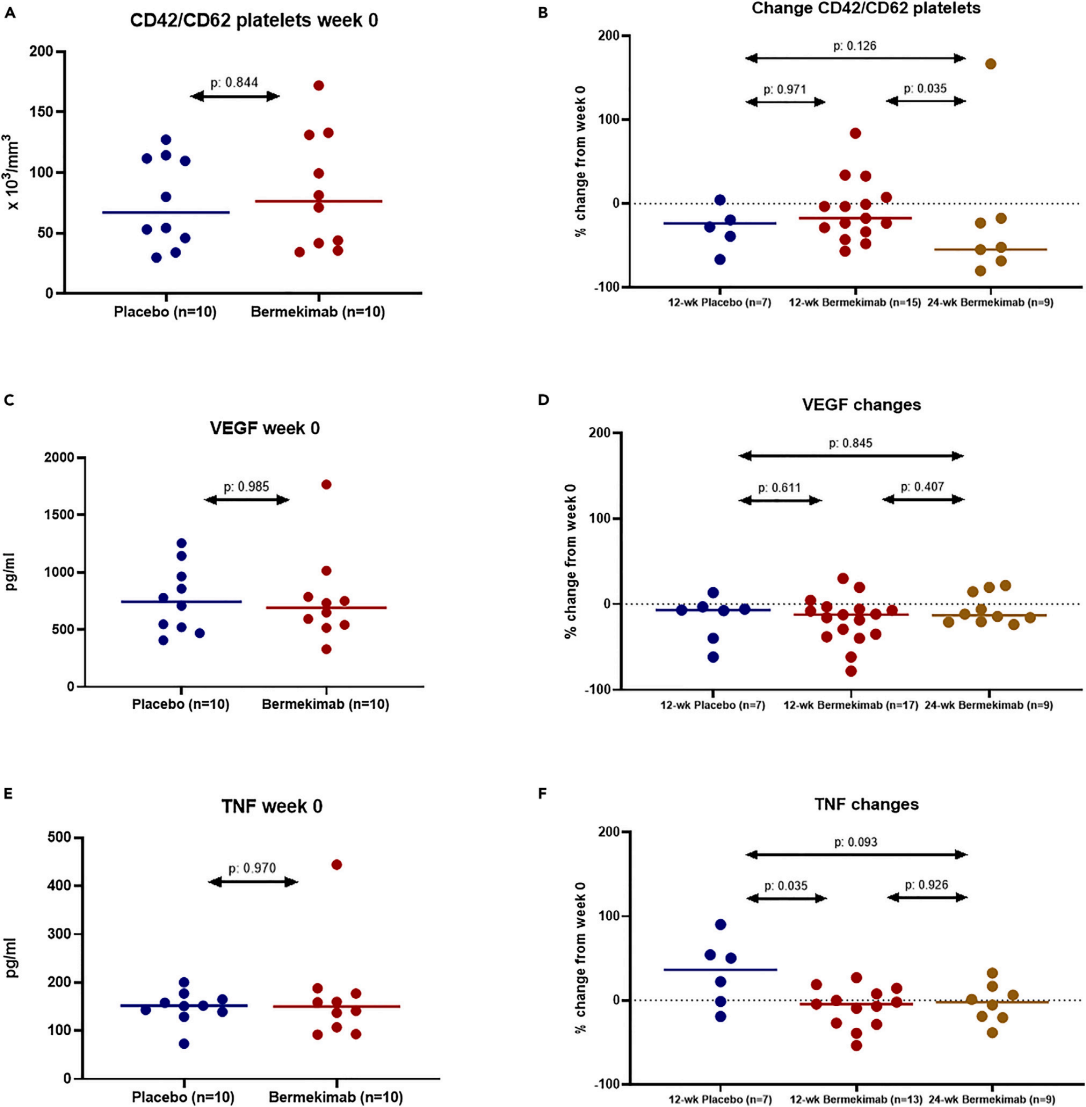
Main biomarker changes following treatment with bermekimab (see also Figure S2) (A and B) Changes of the absolute counts of CD42/CD62 activated platelets over treatment. Panel A shows the absolute counts of CD42/CD62 activated platelets at baseline week 0. Panel B shows the relative changes of the absolute counts of CD42/CD62 activated platelets between patients treated with placebo for 12 weeks, patients treated with bermekimab for 12 weeks and patients treated with bermekimab for 24 weeks. p values of the indicated comparisons are shown. (C and D) Changes of the circulating concentrations of vascular endothelial growth factor (VEGF) over treatment. Panel C shows the concentrations of circulating VEGF at baseline week 0. Panel D shows the relative changes of circulating VEGF between patients treated with placebo for 12 weeks, patients treated with bermekimab for 12 weeks and patients treated with bermekimab for 24 weeks. p values of the indicated comparisons are shown. (E and F) Changes of the circulating concentrations of tumor necrosis factor (TNF) over treatment. Panel E shows the concentrations of cirulating TNF at baseline week 0. Panel D shows the relative changes of circulating TNF between patients treated with placebo for 12 weeks, patients treated with bermekimab for 12 weeks and patients treated with bermekimab for 24 weeks. p values of the indicated comparisons are shown. Abbreviations n: number of patients; wk: weeks.

Although at baseline the biomarker levels were similar between the two groups, two major changes from the baseline were observed: the absolute count of platelets expressing CD42/CD62 was significantly reduced in the bermekimab-treated group and the circulating concentrations of tumor necrosis factor (TNF) were decreased as well (Figure 2). No effects on the kinetics of vascular endothelial growth factor (VEGF), P-Selectin, NT pro-brain natriuretic peptide (BNP), endothelin, creatine phosphokinase (CPK), IL-6 and C-reactive protein were noted (Figure S2).

**Figure 3:**
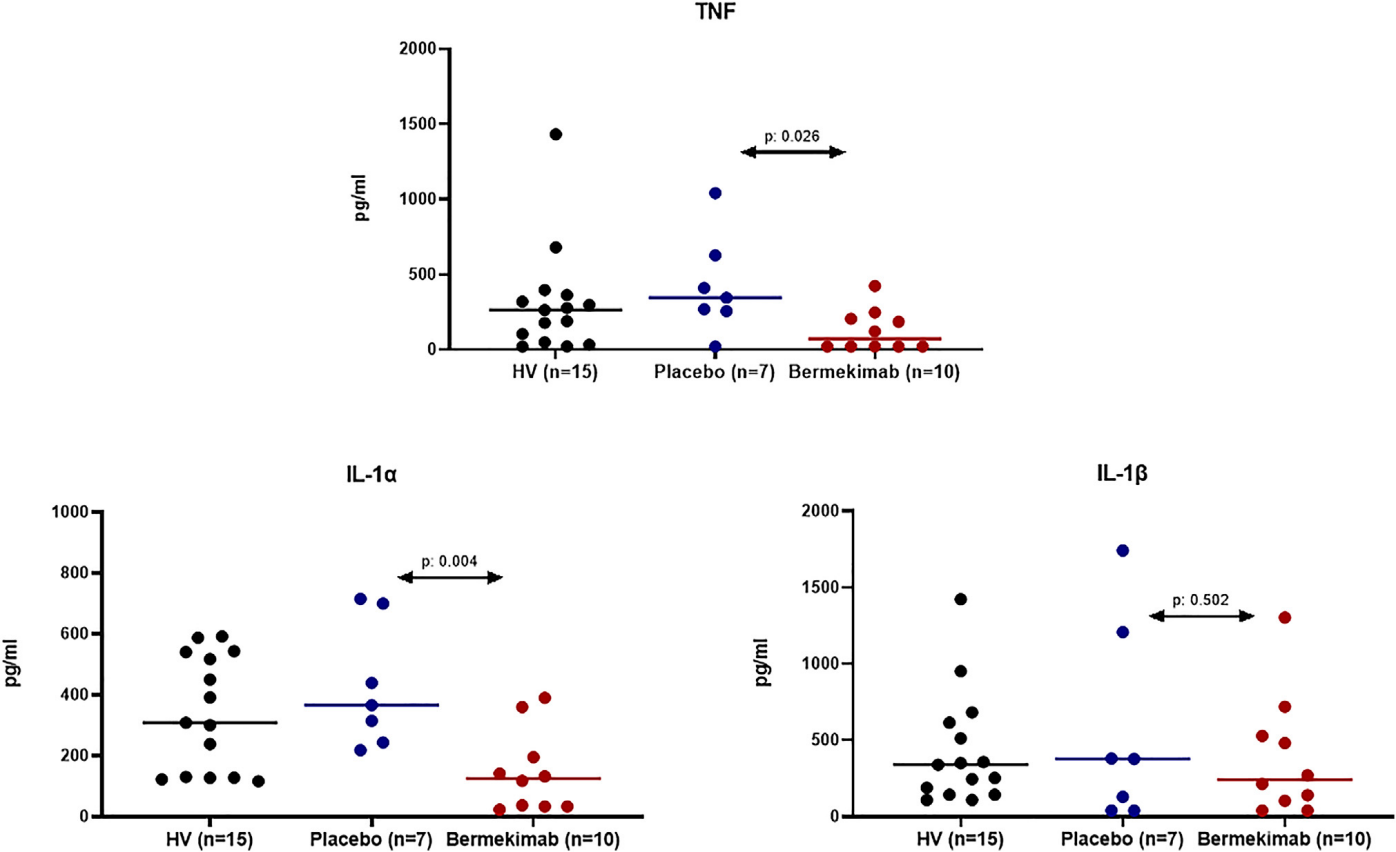
Cytokine production from circulating peripheral blood mononuclear cells (PBMCs) at week 12 PBMCs were isolated after 12 weeks from start of treatment with placebo or bermekimab and stimulated by heat-killed *Staphylococcus aureus* for the production of tumor necrosis factor (TNF), interleukin (IL)-1α and IL-1β. Cytokine production from healthy volunteers (HV) is also provided. p values refer to comparisons indicated by the arrows.

Bermekimab treatment decreased the production of TNF and of IL-1α, but not of IL-1β, from stimulated PBMCs of patients compared to patients treated with placebo (Figure 3).

### Safety

Only one serious treatment-emergent adverse event (TEAE) was reported during the study and this was the case of one patient allocated to placebo treatment who presented with pneumonia and bacteremia; the event ended in death. The incidence of non-serious TEAEs was similar between the two groups with a trend, albeit non-significant, for greater injection site reactions in the bermekimab group (Table 3).

**Table 3:** List of serious and non-serious treatment-emergent adverse events

	Placebo (n = 10)	Bermekimab (n = 10)	p value
Serious treatment-emergent adverse events, n (%)	1 (10)	0 (0)	1.00
Hospitalization for worsening of SSc	1 (10)	0 (0)	1.00
Lower respiratory tract infection and bacteremia	1 (10)	0 (0)	1.00
Death	1 (10)	0 (0)	1.00

**Non-serious treatment-emergent adverse events, n (%)**			

Worsening of SSc	1 (10)	1 (10)	1.00
Flu-like syndrome	0 (0)	1 (10)	1.00
Pyoderma gangrenosum	1 (10)	0 (0)	1.00
Grade 1 blood pressure increased	2 (20)	2 (20)	1.00
Grade 2 blood pressure increased	2 (20)	2 (20)	1.00
Grade 3 blood pressure increased	0 (0)	1 (10)	1.00
Injection site reaction	0 (0)	3 (30)	0.211
Sinus bradycardia	1 (10)	0 (0)	1.00
Sinus tachycardia	1 (10)	0 (0)	1.00
Grade 1 γ-glutamyl transpeptidase increase	1 (10)	0 (0)	1.00
Grade 2 decrease of lymphocytes	0 (0)	1 (0)	1.00

## Discussion

LIGHT is the first randomized clinical trial that provides evidence on the benefit coming from blocking IL-1α for SSc. Most of clinical efficacy was found in decreasing the number of inflamed joints and in attenuating the interstitial lung damage as suggested by the increase of DL_CO_. A trend toward an increase of FVC was also shown, which implies the need to study more patients. The post-hoc confirmation using the CRISS index and the revised CRISS index which incorporates the changes of mRSS, FVC, physician assessment and patient reported outcomes strengthens the conclusion that bermekimab achieved overall clinical improvement. CRISS index and revised CRISS index also validated the primary endpoint of the LIGHT trial.

Patients with SSc have high generation of thrombin^17^ which in turn cleaves pro-IL-1α at the cell membranes of platelets and macrophages to the active moiety.^18^ IL-1α is also produced by the fibroblasts up-stream to the production of IL-6 and PDGF-A; silencing of IL-1α mRNA down-regulates IL-6 and PDGF-A production. IL-6 on its turn stimulates the production of procollagen from fibroblasts,^19^ so it is anticipated that inhibition of IL-1α may have anti-fibrotic effects. Skin keratinocytes, bone chondroblasts and type 2 lung epithelial cells are also foci of IL-1α production which is driving the manifestations of SSc in the joints, skin and lung vasculature.^20^

In the LIGHT trial, a major part of bermekimab efficacy was the improvement of the lung function, as shown by the increase of DLco and the trend toward increase of FVC. This benefit in the lung function is supported indirectly by the measurements of IL-1β and of IL-1ra (receptor antagonist) in the epithelial lining fluid of 54 patients with idiopathic pulmonary fibrosis (IPF). The ratio of IL-1ra to IL-1β ratio was decreased showing patient deficit for the anti-IL-1 responses provided by IL-1ra.^21^ The importance of IL-1α as mediator of lung inflammation has recently been shown in the randomized clinical trial SAVE-MORE; patients with pneumonia by the novel coronavirus SARS-CoV-2 were treated for 10 days with anakinra which is blocking the biological action of both IL-1α and IL-1β. Anakinra treatment decreased by 64% the likelihood of worse outcome compared to placebo as this is expressed by the WHO clinical progression scale.^22^ In the CAN-COVID trial, canakinumab treatment which inhibits the action of only IL-1β did not produce any benefit.^23^

Tissue macrophages is another focus of aberrant IL-1α production in SSc.^24^ Our data show that bermekimab treatment decreased the potential of circulating PBMCs for the production of IL-1α and TNF. The production of IL-1β remained, as anticipated, unaltered due to the lack of activity of bermekimab on IL-1β. The decrease of TNF production by PBMCs is reflected on the decrease of circulating TNF. Similar modulation of the production of IL-1α from whole-stimulated blood has been shown in patients with HS following bermekimab treatment.^12^ IL-1α and NF-κΒ (nuclear factor-kappa B) induce each other in a positive feedback loop.^25^ It is probable that IL-1α blocking antibodies attenuate this mutual induction of IL-1α and NF-κB with a net effect the reduction of IL-1α expression.^26^

Bermekimab treatment effectively decreased the counts of activated platelets. Platelet activation and interaction with the vascular endothelium are hallmarks of the pathogenesis of SSc driving pulmonary arterial hypertension, interstitial edema and impairment of diffusing capacity.^27^^,^^28^ This interaction primes the production of VEGF from the endothelial vasculature which is found increased in the circulation.^28^ In the LIGHT study, patients receiving bermekimab treatment experienced less platelet activation. Circulating concentrations of VEGF were not altered and the effect on platelets was shown after 24 weeks of treatment. This makes a direct effect of bermekimab on the platelet activation cascade questionable.

Results support that the minimum time of treatment required for the clinical benefit to be shown is 12 weeks. This is evidenced by: (1) the superiority of bermekimab over placebo at the end of the 12-week blind period and (2) the clinical benefit of patients originally allocated to placebo when they shifted to bermekimab during the OLE period. Benefit was maintained until week 48. However, the reported efficacy at weeks 36 and 48 was done post-hoc and it did not capture all the 14 elements of the score of inhibition of SSc progression. Another small-scale RCT was performed to investigate the activity of rilonacept (IL-1-trap) in patients with SSc. IL-1-trap treatment, which inhibits both IL-1α and IL-1β failed to meet the main endpoints which involved changes in one 2-gene score and of circulating IL-6 and CCL18.^29^ IL-1-trap also blocks IL-1ra and this may explain, at least in part, the discrepancy between that RCT and the results of the LIGHT trial.

Bermekimab has been studied in 13 other trials including a large diversity of diseases like metastatic colon carcinoma, lung carcinoma, hematologic malignancies, atopic dermatitis and HS. In 12 trials the drug was administered intravenously; it was administered subcutaneously in another open label trial in patients with HS.^30^ One overall safety analysis was provided in the investigated brochure accompanying the dossier submitted in January 2019 to the National Ethics Committee of Greece. Safety was reported for 911 patients treated with bermekimab. The most commonly reported serious TEAEs were infections (5.2%); abdominal pain (1.9%); and anemia (1.5%).^31^

In summary, LIGHT is a randomized clinical trial suggesting that blocking IL-1α with bermekimab may be a promising new strategy for the treatment of SSc. Bermekimab treatment was well tolerated and was associated with monocyte modulation of cytokine production and with attenuation of platelet activation. Further clinical trials with larger number of patients are needed to investigate if bermekimab treatment should be given for only 12 weeks or more and the contribution of prolonged treatment to clinical stability.

### Limitations of the study

Two main limitations of the study need to be addressed: the low number of study participants and the use of CRISS index and of modified CRISS index as post-hoc validation of the study primary endpoint.

## STAR★Methods

### Key resources table

**Table:** 

REAGENT or RESOURCE	SOURCE	IDENTIFIER
**Antibodies**		

PE Mouse Anti-Human CD62P	BD Pharmingen	Cat# 555524; RRID: AB_10547038
PE-Cy5 Mouse Anti-Human CD42b	BD Pharmingen	Cat# 551141

**Chemicals, peptides, and recombinant proteins**		

HBSS	Gibco	14025–092
Fixative Solution	Beckman Coulter	A07800
RPMI 1640 W/Stable Glutamine W/25 MM HEPES	SigmaAldrich	R0833
Lymphosep, Lymphocyte Separation Media	Biowest	L0560
PBS Dulbecco’s Phosphate Buffered Saline w/o Magnesium, w/o Calcium	Biowest	L0615
FBS Superior; standardized Fetal Bovine Serum, EU-approved	Biochrom	S0615
Gentamycin Sulfate BioChemica	PanReac AppliChem	A1492
Penicillin G Potassium Salt BioChemica	PanReac AppliChem	A1837
Adenosine 5′-diphosphate ≥95% HPLC, (ADP)	SIGMA	01905

**Critical commercial assays**		

Human TNF-α uncoated ELISA Kit	Diaclone	EL:1100-34
Human sE-selectin Platinum ELISA Kit	Affymetrix eBioscience	BMS205CE
Human IL-6 ELISA Kit	Diaclone	EL:1006-31
VEGF-A Human Platinum ELISA Kit	Affymetrix eBioscience	BMS277/2CE
Human IL-38/IL-1F 10 Elisa Kit	R&D Systems	DY9110-05
Human EDN1(Endothelin-1) ELISA Kit	Biotech Co	EH0648
Human IL-1 beta uncoated ELISA Kit	Invitrogen	88–7261
Human IL-1 alpha/IL-1F1 ELISA Kit	R&D Systems	DLA50
C-reactive protein	SIEMENS	ADVIA 1800/ADVIA 2400 (Turbidimetric)
Creatine phosphokinase	SIEMENS	ADVIA 1800/ADVIA 2400 (Turbidimetric)
NT pro-BNP	SIEMENS	ADVIA 1800/ADVIA 2400 (Turbidimetric)

**Software and algorithms**		

SPSS	IBM	https://www.ibm.com/analytics/spss-statistics-software

### Resource availability

#### Lead contact

Further information and requests for resources and reagents should be directed to and will be fulfilled by the lead contact, Evangelos J. Giamarellos-Bourboulis (egiamarel@med.uoa.gr).

#### Materials availability

This study did not generate new unique reagents.

### Experimental model and study participant details

LIGHT was a prospective double-blind, placebo-controlled randomized clinical trial that was conducted between June 2019 and February 2021 in two Out-Patient departments in Greece (EudraCT number 2018-004655-20; Approval 8582/1-4-2019 by the National Ethics Committee of Greece; approval 8579/30-5-2019 by the National Organization for Medicines of Greece). All procedures and experiments conform to the relevant regulatory standards. Patients were screened after written informed consent. Participants were adults of Caucasian origin and of either gender who were classified into SSc according to the criteria of the American College of Rheumatology (ACR) and of the European League Against Rheumatism (EULAR); and who had modified Rodnan Skin Score (mRSS) between 15 and 40. For a detailed list of inclusion criteria see Supplement. Exclusion criteria were: age less than 18 years; denial to consent; pregnancy or lactation; renal crisis by SSc; major surgery the last 4 weeks prior to screening; latent tuberculosis; chronic infection by the human immunodeficiency virus (HIV); primary immunodeficiencies; hepatic dysfunction; active bacterial infection; active solid or hematologic malignancy; and malabsorption requiring total parenteral nutrition and neutropenia.

### Method details

The study had a screening period of 0–28 days; one blind period (period A) of 12 weeks; and one open-label extension (OLE, period B) of 12 weeks (Figure 1B). During screening, patients’ eligibility was confirmed after thorough inspection of all clinical information including demographics, comorbidities, medical treatment and disease duration; of SSc-related antibodies; and of clinical assessment of ACR and EULAR classification criteria for musculoskeletal, skin, gastrointestinal and lung manifestations of the disease. Screening involved blood sampling for complete blood cell counts, liver biochemistry and serum creatinine; serology for HIV; and interferon-gamma releasing assay. For women of childbearing potential, a urine pregnancy test was obtained.

During the blinded period A, patients were randomized to treatment for 12 weeks (week 0 to week 11) with subcutaneous placebo or 400 mg bermekimab once every week. During period B, all patients shifted to open-label subcutaneous treatment with 400 mg bermekimab once every week from week 12 to week 23. The duration of treatment was selected based on data of safety coming from the 12-week treatment in HS.^12^^,^^32^ On weeks 0, 12 and 24, complete clinical assessment of SSc was done including the number of inflamed joints; the number of digital ulcers; mRSS; body mass index (BMI); and capillary density by nailfold capillary microscopy (NCM). NCM was graded as 0 (normal), 1 (mild), 2 (moderate) and 3 (severe). Patients were then asked to complete self-questionnaires i.e., gastrointestinal symptoms by University of California Los Angeles Gastrointestinal Tract (UCLA-GIT) scoring system^33^; the quality-of-life using the Short Form questionnaire-36 (SF-36)^34^; their global perception of SSc using one visual analogue scale (VAS) from 0 (best ever felt) to 100 mm (worst ever felt); their perception of fatigue using one VAS from 0 (no fatigue) to 100 mm (worst fatigue ever felt); and their perception of dyspnea using one visual analogue scale (VAS) from 0 (absence) to 100 mm (worst ever felt).

On the same visits (weeks 0, 12 and 24), participants underwent pulmonary function tests of carbon monoxide diffusing capacity (DL_CO_) and forced vital capacity (FVC). DLco was performed with the method of a single breath technique of carbon monoxide uptake (Body Box MedGraphics Elite Series TM Plethysmograph). Participants were also subject to echocardiogram (GE healthcare Vivid S5 cardiovascular ultrasound system) for the estimation of the left ventricle ejection fraction (LVEF) and the pulmonary artery pressure. Echocardiogram was done by placing patients in the left lateral recumbent position using multiple views through the left parasternal and apical windows. Laboratory safety was also monitored on the same visits through determining complete whole blood cell counts, liver biochemistry, international normalized ratio (INR) and serum urea and creatinine. The following exploratory analysis of biomarkers was performed: a) measurements of CRP, TNF, IL-6, VEGF, NT pro-BNP, P-Selectin, endothelin and CPK in plasma; b) isolation of peripheral blood mononuclear cells (PBMCs) and stimulation for the production of TNF, IL-1α and IL-1β; and c) whole blood flow cytometry was done for the expression of the activation markers CD62P (P-selectin) and CD42 (glycoprotein Ib) on platelets.

The study drug and matching placebo were provided as pre-filled syringes of 2mL containing either bermekimab or placebo (XBiotech, Austin, Texas). The content was identical in appearance and blinding was performed by the manufacturers. Separate blind allocation was generated for each study site. Every enrolled participant was assigned a kit containing 12 syringes and each kit had a different number to be used during period A. Every kit was accompanied by a second kit containing 12 syringes of bermekimab to be used during the OLE period B. Each syringe was labeled with a label reading the patient number and the corresponding visit. If a patient was eligible for study enrollment after screening, he/she was assigned one kit number by an electronic randomization system. All investigators were blinded to the study drug. Patients were taught for subcutaneous self-injection in the thigh, arm or abdomen. On each visit, patients were delivered the empty syringes for drug accountability and received syringes in a cooling transport media.

The primary endpoint was the score of inhibition of SSc progression. This was a composite score that contained 14 elements of evaluation coming from the integration of endpoints suggested to be captured in previous SSc trials (Table S2). Patients scoring positive for at least 4 of the 14 elements were considered to achieve inhibition of SSc progression. This primary endpoint was evaluated at week 12 i.e.,.one week after the last dose of phase A and before receiving the first drug injection of the OLE period B. Patients discontinuing the study drug at the discretion of the attending physicians and starting another biological disease modifying anti-rheumatic drug (bDMARD) were considered to fail the primary study endpoint.

Secondary study endpoints were: the positive score of inhibition of SSc progression at week 24; the change of the score of inhibition of SSc progression at week 24 upon shift from placebo to open bermekimab; and the change of each of the 14 elements of the score at week 12 between the two groups of treatment. The evaluation for week 24 was done one week after the completion of the last dose of the OLE period B. Exploratory study endpoints were the comparative kinetics of measured biomarkers during the study between the two groups of treatment.

All patients were monitored throughout the study period for treatment-emergent adverse events (TEAEs) and serious TEAEs.

The study was powered for the primary endpoint with the hypothesis that the score of inhibition of SSc progression will be achieved among 10% of placebo-treated patients and 70% of bermekimab-treated patients. To demonstrate this with 80% power at the 10% level of significance, 10 patients were enrolled in each arm.

Concentrations of TNF and IL-6 (Diaclone, Paris, France), IL-1α (R&D Systems) IL-1β (Invitrogen, Vienna, Austria), VEGF, P-Selectin, (Affymetrix, Vienna, Austria) and endothelin (Biotech Co, Wuhan, China) were measured by an enzyme immunosorbent assay. NT pro-BNP, creatine phosphokinase (CPK) and C-reactive protein (CRP) were measured in ADVIA 2400, SIEMENS, Clinical Chemistry System respectively. The lower limits of detection were: CRP 0.5 mg/L; CPK 1 U/l; NT-proBNP 35 pg/mL; TNF 20 pg/mL, IL-1α 20 pg/mL; IL-1β 40 pg/mL; IL-6 for 31 pg/mL; VEGF 156 pg/mL; P-Selectin 1.6 ng/mL; and 13 ng/ml for endothelin.

Following incubation without/with adenosine biphosphate, whole blood was further incubated for 15 min in the dark with the monoclonal antibodies anti-CD62P FITC (Beckman Coulter, Marseille, France; emission 525mm) and anti-CD42 PE (Beckman Coulter, emission 575mm) and reading through the CYTOMICS FC500 flow cytometer (Beckman Coulter Co, Miami, Florida). Absolute counts were measured using beads.

*Staphylococcus aureus* was used for the stimulation of peripheral blood mononuclear cells (PBMCs) based on previous experience of our group that this stimulation facilitates IL-1α production.^12^ PBMCs were isolated after gradient centrifugation over Ficoll (Biochrom, Berlin, Germany) for 20 min at 1400g. After three washings in ice-cold PBS pH 7.2, PBMCs were counted in a Neubauer plate with trypan blue exclusion of dead cells. They were then diluted in RPMI 1640 enriched with 2mM of L-glutamine, 500 μg/mL of gentamicin, 100 U/ml of penicillin G, 10 mM of pyruvate, 10% fetal bovine serum (Biochrom) and suspended in wells of a 96-well plate. The final volume per well was 200μL with a density of 5 x10^6^ cells/ml. PBMCs were exposed in duplicate for 24 h at 37°C in 5% CO_2_ with 5x10^5^ cfu/ml of heat-killed *Staphylococcus aureus*. At the end of the incubation, cells were centrifuged and supernatants were collected for measurements of TNF and IL-1β by the immunoassay stated above. The cell pellet was treated with Triton X and IL-1α was measured in supernatants by the immunoassay stated above.

### Quantification and statistical analysis

The baseline comparisons between the two groups of treatment were done by the Fisher exact test for qualitative data and by the Mann Whitney U test for quantitative data. The comparison between the two groups of treatment for the primary and secondary endpoints were done by the Fisher exact test; odds ratios (ORs) and 95% confidence intervals (CIs) were calculated according to Mantel and Haenszel’s statistics. Relative changes from baseline in each group were calculated and compared by the Mann Whitney U test. Forward stepwise logistic regression analysis was done using the primary endpoint as dependent variables, group of treatment as independent variables and all different baseline variables between the two groups of treatment as independent variables. In order to validate the score of inhibition of SSc progression, we used measured CRISS (clinical response index of diffuse SSc) index and the revised CRISS index.^15^^,^^16^ For the measurement of the CRISS index, the health assessment questionnaire disability index (HAQ-DI) was completed retrospectively using the answers provided by the patients for the completion of SF-36. The CRISS index was compared between achievers and non-achievers of the score of inhibition of SSc progression and between patients allocated to treatment with placebo and bermekimab by the Mann Whitney U test. Comparisons between the two groups of treatment for the revised CRISS index were done by ordinal regression analysis, calculating ORs and 95% CIs. Relative changes of circulating cytokines and mediators at weeks 12 and 24 from baseline were expressed separately for patients treated the first 12 weeks with placebo; for patients treated for the first 12 weeks with bermekimab by merging patients treated for 12 weeks at the blind period and patients treated for 12 weeks at the OLE period; and for patients treated for the entire study for 24 weeks with bermekimab. Comparisons were done by the Kruskal-Wallis test with Bonferroni corrections for multiple comparisons. Any p value lower than 0.05 was considered statistically significant.

### Additional resources

The LIGHT trial is registered at EudraCT number 2018-004655-20; and at Clinicaltrials.gov NCT04045743.

## Data Availability

• All study data are available from the lead contact (egiamarel@med.uoa.gr) upon request. • This paper does not report original code. • Any additional information required to reanalyze the data reported in this work paper is available from the lead contact (egiamarel@med.uoa.gr) upon request.
