# Supplementary material for: A randomized clinical trial of bermekimab treatment for clinical improvement of systemic sclerosis
Source: iScience. 2023 Aug 19;26(9):107670. doi: 10.1016/j.isci.2023.107670 (PMC10481358; doi:10.1016/j.isci.2023.107670)
Supplement: Document S1. Figures S1 and S2 and Tables S1–S7 [file mmc1.pdf]

iScience, Volume *26*

Supplemental information

A randomized clinical trial of bermekimab treatment for clinical improvement of systemic sclerosis

Nicky Solomonidi, Panayiotis G. Vlachoyiannopoulos, Maria Pappa, Georgia Liantinioti, Sofia Ktena, Evangelos Theotikos, Antonia Elezoglou, Mihai G. Netea, and Evangelos J. Giamarellos-Bourboulis

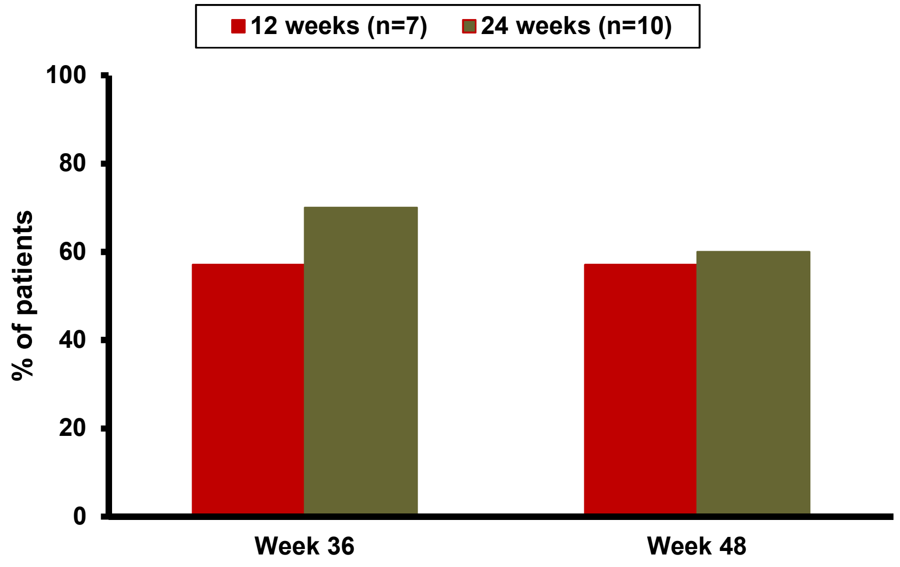

Figure S1 Maintenance of clinical response by weeks 36 and 48 (related to Table 2)
Response to the study drug is provided at weeks 36 and 48 from the start of the study drug. In the Figure, positive response to treatment at weeks 36 and 48 is evaluated post hoc. For weeks 36 and 48, not all 14 elements of the score of inhibition of SSc were captured since the protocol activities were stopped. Patients at weeks 36 and 48 were considered as maintaining positive clinical response provided that they had positive score of inhibition of SSc at week 24 and in addition a) no new drug was started; and b) they were either experiencing the same degrees of joint involvement, fatigue and change of modified Rodnan Skin score (mRSS) as in week 24 or subjective improvement in at least one of joint involvement, fatigue and change of mRSS.

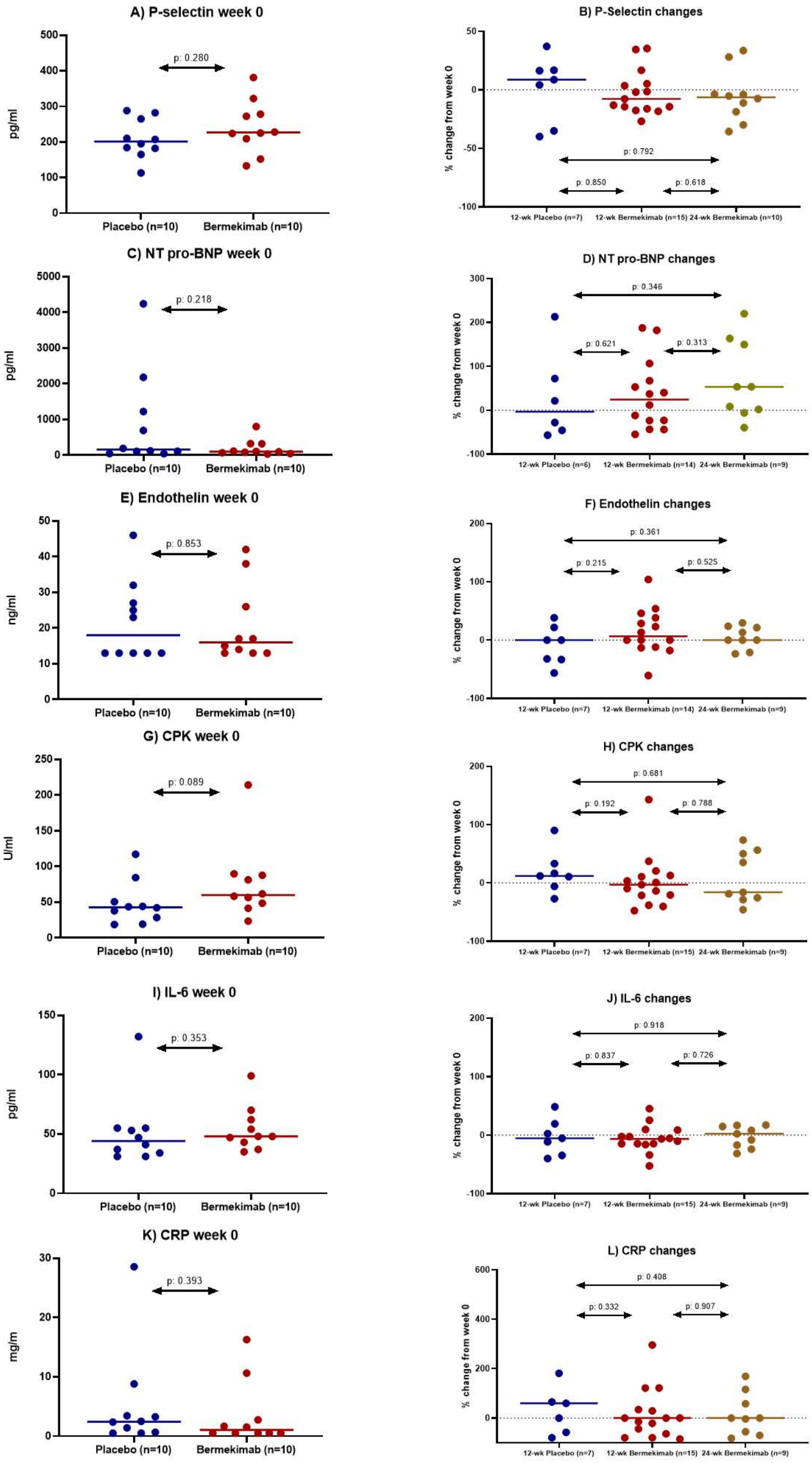

Figure S2 Biomarkers changes during the study (related to Figure 2)
A and B) Changes of circulating P-selectin over treatment. Panel A shows the concentrations of P-selectin at baseline. Panel B shows the relative changes of P-selectin between patients treated with

placebo for 12 weeks, patients treated with bermekimab for 12 weeks and patients treated with bermekimab for 24 weeks. P values of the indicated comparisons are shown.
C and D) Changes of serum NT pro-BNP (brain natriuretic peptide) over treatment. Panel C shows the concentrations of NT pro-BNP at baseline. Panel D shows the relative changes of P-selectin between patients treated with placebo for 12 weeks, patients treated with bermekimab for 12 weeks and patients treated with bermekimab for 24 weeks. P values of the indicated comparisons are shown.
E and F) Changes of circulating endothelin over treatment. Panel E shows the concentrations of endothelin at baseline. Panel F shows the relative changes of P-selectin between patients treated with placebo for 12 weeks, patients treated with bermekimab for 12 weeks and patients treated with bermekimab for 24 weeks. P values of the indicated comparisons are shown.
G and H) Changes of serum Creatinine phosphokinase (CPK) over treatment. Panel G shows the concentrations of CPK at baseline. Panel H shows the relative changes of P-selectin between patients treated with placebo for 12 weeks, patients treated with bermekimab for 12 weeks and patients treated with bermekimab for 24 weeks. P values of the indicated comparisons are shown.
I and J) Changes of circulating interleukin (IL)-6 over treatment. Panel I shows the concentrations of IL-6 at baseline. Panel J shows the relative changes of IL-6 between patients treated with placebo for 12 weeks, patients treated with bermekimab for 12 weeks and patients treated with bermekimab for 24 weeks. P values of the indicated comparisons are shown.
K and L) Changes of serum C-reactive protein (CRP) over treatment. Panel K shows the concentrations of CRP at baseline. Panel L shows the relative changes of IL-6 between patients treated with placebo for 12 weeks, patients treated with bermekimab for 12 weeks and patients treated with bermekimab for 24 weeks. P values of the indicated comparisons are shown.
Abbreviations n: number of patients; wk: weeks

Table S1 Affected joints among patients without synovitis at baseline (related to Table 1)

	**Placebo (n=7)**	**Bermekimab (n=7)**	**P-value**
Affected join areas, n (%)			
Left shoulder	4 (57.1)	2 (28.6)	0.582
Left elbow	3 (42.9)	3 (42.9)	1.00
Left carpal	5 (71.4)	6 (85.7)	1.00
Left MCP	4 (57.1)	3 (42.9)	1.00
Left hip	1 (14.3)	1 (14.3)	1.00
Left knee	2 (28.6)	2 (28.6)	1.00
Left ankle	1 (14.3)	1 (14.3)	1.00
Right shoulder	4 (57.1)	2 (28.6)	0.582
Right elbow	3 (42.9)	4 (57.1)	1.00
Right carpal	5 (71.4)	6 (85.7)	1.00
Right MCP	3 (42.9)	3 (42.9)	1.00
Right hip	1 (14.3)	1 (14.3)	1.00
Right knee	2 (28.6)	2 (28.6)	1.00
Right ankle	1 (14.3)	2 (28.6)	1.00

Abbreviations MCP: metacarpophalangeal

Table S2 The 14-point score of inhibition of progression of Systemic Sclerosis (SSc) (related to Figure 1)
The score delivers one point for each element. Patients meeting at least four of the elements are considered to have achieved inhibition of SSc. The column next to the element denotes the publication from which the element has derived.

	**Reporting Reference**
At least 30% decrease of the number of inflamed joints	[S1]
At least 30% decrease of the number of digital ulcers	[S2]
At least 30% decrease of the mRSS	[S3]
At least 50% decrease of the UCLA GIT scoring system	[S4]
At least 50% increase of the SF-36	[S5]
At least 50% decrease of VAS for SSc	[S6]
At least 50% decrease of VAS for fatigue	[S6]
At least 50% decrease of VAS for dyspnea	[S6]
Any increase of body mass index	[S7]
Any increase of carbon monoxide diffusing capacity (DL_{CO})	[S8]
Any increase of forced vital capacity (FVC)	[S8]
At least 10% increase of the left ventricle ejection fraction (LVEF)	[S9]
At least 10% decrease of the pulmonary artery pressure	[S10]
At least 10% decrease of the capillary density as assessed by NCM	[S11]

Abbreviations mRSS: Modified Rodnan Skin Score; UCLA GIT: University of California Los Angeles Gastrointestinal Tract questionnaire; SF-36: short form 36 health survey; VAS: visual analogue scale NCM; nailfold capillaromicroscopy

Table S3 Changes of each variable of the primary endpoint from baseline (related to Table 2)

Variable	Bermekimab (n=10)	Placebo (n=10)
Relative change of the number of inflamed joints from baseline, %, median (IQR)	-37.9 (50)	0 (62.5)
Relative change of modified Rodnan Skin Score from baseline, %, median (IQR)	-11.6 (12.3)	-13.7 (24.1)
Relative change of the number of digital ulcers from baseline, %, median (IQR)	-50.0 (85.0)	0 (75.0)
Relative change of UCLA-GIT from baseline, %, median (IQR)	10.7 (87.1)	0 (122.1)
At least 50% increase of the short form 36 health survey	3 (30)	4 (40)
Relative change of global VAS from baseline, %, median (IQR)	-17.1 (46.1)	0 (27.1)
Relative change of VAS for fatigue from baseline, %, median (IQR)	-5.6 (51.1)	0 (28.8)
Relative change of VAS for dyspnea from baseline, %, median (IQR)	-12.5 (58.3)	-5.0 (41.7)
Relative change of SF-36v for dyspnea from baseline, %, median (IQR)	25.1 (88.5)	36.5 (59.2)
Relative change of body mass index from baseline, %, median (IQR)	0 (1.7)	0 (2.5)
Relative change of nailfold capillaromicroscopy from baseline, %, median (IQR)	0 (0)	0 (0)
Relative change of carbon monoxide diffusing capacity from baseline, %, median (IQR)	2.5 (18.3)	-1.4 (4.3)
Relative change of forced vital capacity from baseline, %, median (IQR)	0.6 (8.9)	0 (4.4)
Relative change of left ventricular ejection fraction from baseline, %, median (IQR)	0 (2.3)	9 (0)
Relative change of pulmonary arterial pressure from baseline, %, median (IQR)	-2.7 (27.4)	0 (2.2)

Abbreviations IQR: interquartile range; UCLA GIT; University of California Los Angeles Gastrointestinal Tract scoring system SF-36: short form 36 health survey; VAS: visual analogue scale

Table S4 Forward step-wise logistic regression analysis to define variables associated with achievement of the primary endpoint (related to Table 2)

Variables entering in the equation are all four baseline variables different between patients allocated to treatment with placebo and to treatment with bermekimab with p-value less than 0.100

Variable	Primary endpoint		Univariate analysis		Multivariate analysis	
	Negative (n=10)	Positive (n=10)	OR (95% CIs)	p-value	OR (95% CIs)	p-value
Bermekimab treatment, n (%)	2 (20)	8 (80)	16.0 (1.8-143.1)	0.013	16.0 (1.8-143.1)	0.013
Age, years, mean (SD)	52.3 (16.0)	48.5 (12.3)	0.97 (0.91-1.04)	0.448	*	
BMI, kg/m^2, mean (SD)	27.1 (6.3)	23.3 (6.2)	0.89 (0.77-1.05)	0.184	*	
mRSS at baseline, mean (SD)	20.1 (3.2)	26.3 (9.6)	1.15 (0.97-1.35)	0.100	*	
Number of digital ulcers at baseline, mean (SD)	1.0 (1.2)	3.2 (3.1)	1.83 (0.96-3.47)	0.066	*	

*variables excluded after one step of forward analysis

Abbreviations BMI: body mass index; CI: confidence interval; mRSS: modified Rodnan skin score; n: number of patients; OR: odds ratio; SD: standard deviation

Table S5 Comparisons of the revised CRISS index between the two groups of treatment (related to Table 2)

	Bermekimab (n= 10)	Placebo (n=10)	OR (95%CIs)	p-value
	Patients with at least 10% performance improvement in one of PGA, VAS, HAQ-DI, mRSS and/or 5% increase of FVC			
Nil improvement, n (%)	0 (0)	5 (50)		
1 improvement, n (%)	1 (10)	0 (0)		
2 improvements, n (%)	2 (20)	1 (1)	5.03 (0.90-27.9)	0.062
3 improvements, n (%)	4 (40)	3 (30)		
4 improvements, n (%)	3 (30)	0 (0)		
5 improvements, n (%)	0 (0)	1 (0)		
	Patients with at least 20% performance improvement in one of PGA, VAS, HAQ-DI, mRSS and/or 5% increase of FVC			
Nil improvement, n (%)	0 (0)	4 (40)		
1 improvement, n (%)	2 (20)	2 (20)	5.69 (1.17-62.9)	0.034
2 improvements, n (%)	7 (70)	4 (40)		
3 improvements, n (%)	1 (10)	0 (0)		
	Patients with at least 30% performance improvement in one of PGA, VAS, HAQ-DI, mRSS and/or 5% increase of FVC			
Nil improvement, n (%)	2 (20)	6 (60)		
1 improvement, n (%)	6 (60)	4 (40)	7.36 (1.03-52.5)	0.046
2 improvements, n (%)	2 (20)	0 (0)		

Abbreviations CI: confidence interval; FVC: forced vital capacity; HAQ-DI: health assessment questionnaire-disability index; mRSS: modified Rodnan skin score; n: number of patients; OR: odds ratio; PGA: physician's global assessment; VAS; visual analogue scale

Table S6 Comparison between patients treated for 24-weeks (allocated to the active arm at phase A of the study) and patients treated for 12-weeks with bermekimab (allocated to the placebo arm at phase A of the study) at the end of the open-label phase B of the study The column of 12-week treatment contains the 10 patients originally treated with bermekimab at the blind phase A and the seven patients originally treated with placebo and switched to bermekimab during the open-label extension phase B. The column of 24-week treatment contains the 10 patients treated with bermekimab during both study periods (related to Table 2)

**Endpoint**	**24 weeks (n=10)**	**12 weeks (n=17)**	**OR (95% CIs)**	***P* value**
Achievement of score of inhibition of SSc progression 4 or more at week 24, n (%)	8 (80)	13 (76.5)	1.23 (0.18-8.33)	1.00
Score of inhibition of SSc progression at week 24, median (Q1-Q3)	6 (3.5-7.0)	5.0 (3-6.0)	N/A	0.863
Positive elements of the score of inhibition of SSc progression, n (%)				
At least 30% decrease of the number of inflamed joints	9 (90)	13 (76.5)	2.77 (0.26-39.01)	0.621
At least 30% decrease of modified Rodnan skin score	7 (70)	5 (29.4)	5.60 (1.01-30.91)	0.056
At least 30% decrease of digital ulcers	5 (50)	8 (47.1)	1.13 (0.24-5.37)	1.00
At least 50% decrease of UCLA-GIT	3 (30)	6 (35.3)	0.78 (0.15-4.21)	1.00
At least 50% increase of the SF-36	3 (30)	7 (41.2)	0.61 (0.12-3.22)	0.691
At least 50% decrease of global VAS	1 (10)	6 (35.3)	0.20 (0.02-2.02)	0.204
At least 50% decrease of VAS for fatigue	2 (20)	4 (23.5)	0.81 (0.12-5.39)	1.00
At least 50% decrease of VAS for dyspnea	2 (20)	6 (35.3)	0.49 (0.08-2.89)	0.666
Any increase of body mass index	4 (40)	6 (35.3)	1.22 (0.24-6.11)	1.00
At least 10% decrease of nailfold capilaromiscoscopy	3 (30)	6 (35.3)	0.79 (0.15-4.21)	1.00
Any increase of carbon monoxide diffusing capacity	6 (60)	6 (35.3)	2.75 (0.55-13/75)	0.256
Any increase of forced vital capacity	3 (30)	6 (35.3)	0.79 (0.15-4.21)	1.00
At least 10% increase of left ventricular ejection fraction	1 (10)	2 (11.8)	0.83 (0.06-10.55)	1.00
At least 10% decrease of pulmonary arterial pressure	5 (50)	6 (35.3)	0.83 (0.06-10.55)	1.00

Abbreviations CI: confidence interval; OR: odds ratio; UCLA GIT: University of California Los Angeles Gastrointestinal Tract questionnaire; SF-36: short form 36 health survey VAS; visual analogue scale

Table S7 Follow-up of the patients after the end of the study (related to Table 2)
Follow-up is provided at weeks (wks) 36 and 48 from study enrolment i.e. 12 weeks and 24 weeks respectively after end of the study drug. Results are provided per patient.
For weeks 36 and 48 not all 14 elements of the score of inhibition of systemic sclerosis (SSc) were captured. Patients were considered as maintaining positive clinical response if that they had positive score of inhibition of SSc at week 24 and in addition a) no new drug was started; and b) they were either experiencing the same degrees of joint involvement, fatigue and change of modified Rodnan Skin score (mRSS) as in week 24 or subjective improvement in at least one of joint involvement, fatigue and change of mRSS.

Number	Randomization	Need for new drug	Type of new drug	Clinical condition	Positive clinical response
1	Placebo	No	None	Stable on negative score of inhibition of SSc compared to wk24	No
2	Placebo	Yes	Pulse corticosteroids	Worsening of arthritis	No
3	Placebo	No	None	Stable on negative score of inhibition of SSc compared to wk24	No
4	Placebo	No	None	Further improvement in arthritis and fatigue compared to wk24	Yes
5	Placebo	No	None	Hospitalization for super-infection of digital ulcers	No
6	Placebo	Yes	Rituximab	Stable on negative score of inhibition of SSc compared to wk24	No
7	Placebo	No	None	Further improvement in arthritis, fatigue and ulcers compared to wk24	Yes
8	Placebo	No	None	Further improvement in arthritis, fatigue, skin elasticity and ulcers compared to wk24	Yes
9	Placebo	No	None	Stable on negative score of inhibition of SSc compared to wk24	No
10	Placebo	No	None	Further improvement in fatigue compared to wk24	Yes
11	Bermekimab	Yes	Nintedanib	Worsening of dyspnea	No
12	Bermekimab	No	None	Further improvement in arthritis and fatigue compared to wk24	Yes
13	Bermekimab	No	None	Stable on positive score of inhibition of SSc compared to wk24	Yes
14	Bermekimab	No	None	Stable on positive score of inhibition of SSc compared to wk24	Yes
15	Bermekimab	No	None	Further improvement in arthritis and skin elasticity compared to wk24	Yes

16	Bermekimab	No	None	Further improvement in fatigue compared to wk24	Yes
17	Bermekimab	No	None	Further improvement in arthritis compared to wk24	Yes
18	Bermekimab	No	None	Stable on negative score of inhibition of SSc compared to wk24	No
19	Bermekimab	No	None	Died	No
20	Bermekimab	No	None	Further improvement in arthritis, fatigue, skin elasticity and ulcers compared to wk24	Yes

REFERENCES

1. Avouac, J., Walker, U., Tyndall, A., et al. (2010). Characteristics of joint involvement and relationships with systemic inflammation in systemic sclerosis: results from the EULAR scleroderma trial and research group (EUSTAR) database. J Rheumatol *37*, 1488-501.
2. Mihai, C., Landewe, R., van der Heijde, D., et al. (2016). Digital ulcers predict a worse disease course in patients with systemic sclerosis. Ann Rheum Dis *75*, 681-6.
3. Khanna, D., Furst, D.E. Clements, P.J., et al. (2017). Standardization of the modified Rodnan skin score for use in clinical trials of systemic sclerosis. J Scleroderma Relat Disord *2*, 11-8.
4. Baron, M., Hudson, M., Steele, R., et al. (2011). Validation of the UCLA scleroderma clinical trial gastrointestinal tract instrument version 2.0 for systemic sclerosis. J Rheumatol *38*, 1925-30.
5. Khanna, D., Furst, D.E. Clements, P.J., et al. (2005). Responsiveness of the SF-36 and the health assessment questionnaire disability index in a systemic sclerosis trial. J Rheumatol *32*, 832-40.
6. Merkel, P.A., Herlyn, K., Martin, R.W., et al. (2002). Measuring disease activity and functional status in patients with scleroderma and Raynaud's phenomenon. Arthritis Rheum *46*, 2410-20.
7. Bishop, V., Harrison, E., Lal, S., et al. (2015). Evidence for a clinical association between body mass index and malabsorption in patients with systemic sclerosis. Scand J Rheumatol *44*, 341-3.
8. Goh, N.S., Hoyles, R.K., Denton, C.P., et al. (2017). Short-term pulmonary function trends are predictive of mortality in interstitial lung disease associated with systemic sclerosis. Arthritis Rheumatol *69*, 1670-8.
9. Spethmann, S., Rieper, K., Riemekasten, G., et al. (2014). Echocardiographic follow-up of patients with systemic sclerosis by 2D speckle tracking echocardiography of the left ventricle. Cardiovasc Ultrasound *12*, 13.
10. Chung, L., Domsic, R.T., Lingala, B., et al. (2014). Survival and predictors of mortality in systemic sclerosis-associated pulmonary arterial hypertension: outcomes from the pulmonary hypertension assessment and recognition of outcomes in scleroderma registry. Arthritis Care Res (Hoboken) *66*, 489-95.
11. Smith, V, Thevissen, K, Trombetta, AC, et al. (2016). Nailfold capillaroscopy and clinical applications in systemic sclerosis. Microcirculation *23*, 364-72.
